# Somatic comorbidity and the progression of cognitive impairment

**DOI:** 10.3389/fnagi.2023.1219449

**Published:** 2023-11-17

**Authors:** Leszek Bidzan, Paweł Jurek, Michał Olech, Monika Bidzan-Wiącek, Ilona Bidzan-Bluma, Mariola Bidzan

**Affiliations:** ^1^Faculty of Medicine, Medical University of Gdańsk, Gdańsk, Poland; ^2^Department of Health Sciences, Pomeranian University in Słupsk, Słupsk, Poland; ^3^Institute of Psychology, University of Gdańsk, Gdańsk, Poland; ^4^Faculty of Health Sciences, Medical University of Gdańsk, Gdańsk, Poland; ^5^Institute of Pedagogy and Languages, University of Applied Sciences in Elbląg, Elbląg, Poland

**Keywords:** cognition, dementia, MCI, comorbidities, neurosciences, MMSE, CDR-SB, activity

## Abstract

**Background:**

There are usually multiple factors underlying dementia in old age. Somatic comorbidity is one important element that influences the progression of cognitive impairment.

**Objective:**

The goal of this study was to assess the relationship between the progression of cognitive impairment and the presence and severity of comorbidities based on a four-year observation.

**Material:**

Out of 128 patients from the Clinic for Outpatients in Gdansk, who were recruited into the study based on the criteria of the Working Group on Mild Cognitive Impairment, a total of 93 participants completed the four-year observation. Only the data from participants who completed the full period of observations were analysed. The mean age of the group was *M* = 75.93 (*SD* = 9.43). The level of progression of cognitive impairment was measured using the Clinical Dementia Rating Scale – Sum of Boxes, the severity of comorbidities was measured using the modified Cumulative Illness Rating Scale, and, additionally, at the time of inclusion in the study, participants were assessed using the MMSE scale and the Activity Scale, and sociodemographic data were collected. The Generalized Estimating Equations method was employed to fit a marginal model for analyzing the data collected in a repeated measures design. The tested model elucidated the role of the overall severity of comorbidities in explaining the progression of cognitive impairment, while controlling for everyday activity and basic demographic variables.

**Results:**

During the four-year observation, a significant decline in cognitive function (*B* = 1.86, *p* < 0.01) was observed in the examined sample. The statistical analysis revealed that individuals with higher overall severity of comorbidities exhibited significantly more pronounced progression of cognitive impairment over time. Regarding particular comorbidities, metabolic diseases were found to be associated with a poorer prognosis (*rho* = 0.41, *p* < 0.05). Furthermore, a time physical activity interaction was identified as predicting cognitive impairment, indicating that individuals who were more physically active at the beginning of the study exhibited significantly less pronounced progression of cognitive impairment over the course of the 4 years.

**Conclusion:**

This study suggests the important roles of comorbidities and physical activity for the prognosis of mild cognitive impairment.

## 1 Introduction

As the mean population age increases, so does the prevalence of dementia; this is already a serious socio-economic problem, being a burden for both healthcare systems and the caregivers of those affected. Dementia is usually preceded by a period of impaired cognitive functioning, which can be of different lengths in different people (Bookheimer et al., 2000; Bidzan and Bidzan, 2014; Cooper et al., 2019; Edmonds et al., 2019). A decrease in cognitive fitness is associated with the risk of onset of fully symptomatic dementia in a relatively short period of time. This risk is especially serious if the impairment deteriorates, taking the form of mild cognitive impairment (MCI) (Bidzan and Bidzan, 2014; Pąchalska et al., 2015; Bidzan et al., 2017; Cooper et al., 2019). MCI is defined as when cognitive function (usually memory) is impaired, but functionality is preserved (i.e., a person with MCI has no significant impairment in their abilities to perform tasks of daily living), in the absence of medical disturbances. The most recent characterization of MCI includes the following criteria: (1) a subjective sense of the presence of memory disorder (2) intact ability to carry out everyday activities, such as meal preparation; (3) preserved general cognitive abilities; (4) objective evidence of a memory deficit; and (5) the absence of dementia (Kempf et al., 2016; Livingston et al., 2020).

The yearly prevalence of onset of dementia in individuals with MCI is significantly higher than among the general population, varying between 4.9% and 9.6% (Mitchell and Shiri-Feshki, 2009; Bidzan et al., 2017). The severity of further progression of cognitive impairment depends on many factors: in addition to age, these also include family history (Fratiglioni et al., 1993; Bidzan et al., 2017; Treder-Rochna, 2020) and genetic factors such as the ε4 allele of Apolipoprotein E (Gylys et al., 2010; Jóźwik et al., 2012; Livingston et al., 2020). However, only genetic factors and age are non-modifiable – other factors can be modified through interventions. This is why efforts should focus on identifying potentially modifiable factors which could accelerate the process of cognitive deterioration. Around 35%–40% of all dementia cases are attributable to modifiable risk factors (Kempf et al., 2016; Livingston et al., 2017, 2020). A growing body of evidence supports the 12 potentially modifiable risk factors for dementia proposed by the 2017 Lancet Commission on dementia prevention, intervention, and care: low education level, hypertension, hearing impairment, smoking, obesity, depression, physical inactivity, diabetes, low social contact, excessive alcohol consumption, traumatic brain injury, and air pollution (Livingston et al., 2020).

However, it needs to be noted that the processes that lead to the histopathological changes which are the basis of dementia begin many years, if not decades, before the clinical onset of the condition (Nutaitis et al., 2019).

Individuals with dementia are found to have many concomitant conditions. On average, such patients have diagnoses of two to eight other conditions (Schubert et al., 2006). These conditions can disrupt a patient’s functioning and make caregiving more difficult (Chen et al., 2018). They may also influence the severity of the progression of dementia-related impairment (Cooper et al., 2015). Epidemiological studies have found that the occurrence of conditions such as hypertension, diabetes, and hyperlipidemia in middle age is associated with a higher risk of Alzheimer’s-type dementia as well as vascular dementia later on in life (Reijmer et al., 2012; Baumgart et al., 2015; Kempf et al., 2016). In addition to cardiovascular factors, conditions of the respiratory system, the liver (cirrhosis), and kidneys may also be risk factors (Chen et al., 2018). While the association between cardiovascular factors in middle age and the risk of dementia seems well documented, their impact at later stages of life requires further study.

## 2 Methods

### 2.1 Study design

In order to investigate the progression of cognitive impairment, a prospective observational study with repeated measures was carried out. Over the course of the four-year study, a psychiatrist observed and analysed a cohort of patients, refraining from intervening or imposing any experimental treatments. The principal aim of the study was to acquire information on the course of cognitive impairment advancement of cognitive impairment and to uncover potential correlations between cognitive impairment and somatic comorbidity assessed at the study’s outset. A battery of tests and a demographic questionnaire were administered during the initial visits, once the participants had consented to engaging in the study. After 4 years, measurement of the outcome variable – cognitive impairment – was repeated.

In this study design, researchers refrain from active interventions, allowing the accumulation of real-world data within a more naturalistic context. Nevertheless, the lack of controlled interventions makes it challenging to establish cause-and-effect relationships, as other confounding variables may influence the observed outcomes (Hammoudeh et al., 2018).

This research project was reviewed and approved by the Bioethics Committee (decision no. NKBBN/279/2014) at the Medical University of Gdansk, Poland.

### 2.2 Inclusion and exclusion criteria

Participants were enrolled in the study based on the following criteria.

#### 2.2.1 Inclusion criteria

(1) Voluntary agreement to participate; (2) diagnosis of amnestic Mild Cognitive Impairment (MCI) according to the Working Group on Mild Cognitive Impairment criteria, i.e., (a) evident cognitive decline self-reported by the participant or reported by a caregiver, confirmed by objective cognitive impairment assessment, and/or observable cognitive decline over time through objective examination (Winblad et al., 2004); (b) ability to perform basic daily activities preserved, with minimal impairment in complex activities; (c) and not currently undergoing psychiatric pharmacotherapy during recruitment; (3) presence of an individual from the participant’s daily environment who lives with them or visits them several times weekly and consents to participate as an informant; (4) global score of 0.5 on the Clinical Dementia Rating (Global CDR); and (5) Mini-Mental State Examination score between 24 and 30 (Folstein et al., 1975).

#### 2.2.2 Exclusion criteria

(1) Diagnosis of dementia, regardless of etiology, according to the DSM-5 (American Psychiatric Association, 2013); (2) current or past diagnosis of any of the following conditions: mood disorders, schizophrenia, alcoholism, drug or substance dependence, epilepsy, Parkinson’s disease, or any intellectual disability; (3) present impaired consciousness, or motor, visual, or hearing impairment significantly hindering the ability to complete tasks and assessments in the clinical scales used in the study; and (4) withdrawal of consent to participate at any stage of the study.

### 2.3 Participants

A cohort of amnestic Mild Cognitive Impairment (MCI) patients was enrolled at the Clinic for Outpatients in Gdansk, Poland. A total of 128 participants with diagnoses of amnestic MCI were eligible for this study. The recruitment for the study lasted from the end of 2015 through 2016, and each individual was observed for the 4 years after their enrolment in the study. All data were collected as a part of a routine clinical visit. We excluded 35 participants who, for various reasons, could not be re-examined after 4 years. Thus, the study sample consisted of 93 participants (71 women and 22 men) with diagnosed MCI. At the start of the study, the average age of the participants was *M* = 75.93 (*SD* = 9.43). They varied in terms of the number of years of formal education (*M* = 11.77, *SD* = 4.25). Over the course of the study, 18 participants received a diagnosis of dementia.

### 2.4 Measures

#### 2.4.1 Primary outcome: progression of cognitive impairment

The Clinical Dementia Rating Scale – Sum of Boxes (CDR-SB) developed by Hughes et al. (1982) was used for evaluating the progression of cognitive impairment. The CDR-SB consists of six items covering the main domains of cognitive functioning: memory, orientation, judgment and problem solving, community affairs, home and hobbies, and personal care. Each domain is rated by an interviewer on a five point behaviorally anchored scale (0 = *no cognitive impairment*, 0.5 = *questionable or very mild dementia*, 1 = *mild dementia*, 2 = *moderate dementia*, and 3 = *severe dementia*). The overall CDR-BS score (ranging from 0 to 18) is obtained by summing each of the six domain scores. In the current study, the CDR-SB scores were collected twice: first during the initial examination and then during the follow-up after 4 years. Both results were based on information collected from the patient and caregiver (informant). The Cronbach’s alpha coefficient for both measurements in the present study was 0.78, indicating a satisfactory level of reliability.

#### 2.4.2 Cognitive functioning: initial assessment

The Mini-Mental State Examination (MMSE), developed by Folstein et al. (1975), Polish adaptation by Stańczak (2010), was used to assess cognitive functioning. The MMSE is a 30-item tool that includes tests of orientation, attention and calculation, memory, language, and visual–spatial skills. Participants’ responses are scored “1” if correct or “0” if incorrect. The Polish version of the MMSE has been shown to have high internal consistency in diverse clinical samples (Stańczak, 2010).

#### 2.4.3 Somatic comorbidity: initial assessment

The modified Cumulative Illness Rating Scale (CIRS) was used to assess somatic comorbidity (Salvi et al., 2008). The modified CIRS includes 15 categories that assess physical impairment. Each category applies to a relatively independent body system and is rated on a five-point scale (from 1 = *no impairment to that organ/system* to 5 = *impairment is life threatening*). However, previous studies have shown that results for the CIRS categories have skewed distributions, with the mode being the lowest value for all items (Cova et al., 2016). Therefore, the items were dichotomized (0 = *no physical impairment*, 1 = *physical impairment diagnosed*), making it less likely to result in biased estimates than more unbalanced scales (Pedhazur and Schmelkin, 1991). The overall CIRS score was obtained by summing each of the 15 individual system scores (using the original values before dichotomization).

#### 2.4.4 Sociodemographic variables and living activities

The participants were interviewed and asked about their number of years of formal education, sex, and age. To assess the participants’ living activities, the Activity Scale (Bidzan et al., 2016) was used, which is our own modification of the scale by Christensen and Mackinnon (1993). It consists of 13 items that measure the intensity of three types of activity: intellectual (five items; e.g., reading books and magazines or doing crosswords), physical (three items; e.g., sports and recreation, such as bicycling, skiing, aerobics, or gymnastics), and social (five items; e.g., playing games or having a good time with others). Each activity was rated on a four-point frequency scale from 0 (*not at all*) to 3 (*very often*). The score for each subscale was computed by summing up the points scored on items that pertain to a given type of activity.

### 2.5 Statistical analyses

We employed the Generalized Estimating Equations (GEE) method as proposed by Liang and Zeger (1986) and Ballinger (2004). This method is particularly suitable for generating regression estimates when dealing with repeated measures involving non-normally distributed outcome variables. The tested model provided insights into the association between overall severity of comorbidities and the progression of cognitive impairment. This analysis was performed while accounting for three categories of daily living activities – intellectual, physical, and social – as well as relevant demographic variables, including age, sex, and years of education. Furthermore, in line with the approach of Wang et al. (2016), we integrated more recent modified variance estimators into the model fitting procedure. This choice was driven by the goal of enhancing the small-sample performance of the model, thereby contributing to the robustness of our findings.

## 3 Results

The main characteristics of the participants’ cognitive functioning and somatic impairment are presented in Table 1. A more in-depth examination of the data revealed that hypertension (38%), along with cardiac (48%) and metabolic (36%) impairment, were prevalent within the studied sample. The frequencies of physical and social activity were moderate; the frequency of intellectual activity was rather low.

**Table 1:** Descriptive statistics for the main variables examined in the study.

Variable	M	SD	Min.	Max.	Skew.	Kurt.
Cognitive functioning (MMSE)	26.77	1.45	24.00	29.00	−0.12	−0.90
Cognitive impairment (CDR-SB) – pre-test	1.73	0.97	0.50	4.50	0.74	0.05
Cognitive impairment (CDR-SB) – post-test	3.59	2.96	0.50	17.00	2.59	8.19
Cognitive impairment progression (ΔCDR-SB)	1.86	2.63	0.00	13.50	2.95	9.56
Cumulative illness – CIRS overall score	19.88	2.51	16.00	26.00	0.54	−0.48
Intellectual activity	2.86	1.74	1.00	6.00	0.57	−0.99
Physical activity	5.68	2.83	1.00	9.00	−0.24	−1.36
Social activity	5.37	3.57	1.00	11.00	0.19	−1.32

M, mean; SD, standard deviation.

The utilization of the Generalized Estimating Equations (GEE) method provided insight into the dynamic nature of cognitive impairment throughout the study period. By accounting for the nature of the repeated measures data, the GEE model was able to identify trends and associations within the dataset. Table 2 shows regression estimates encompassing both primary and controlled variables, elucidating their roles in predicting cognitive impairment.

**Table 2:** Regression estimates for tested models using Generalized Estimating Equations (GEE).

Predictor	Model 1			Model 2			Model 3		
	*B*	*SE*	*p*	*B*	*SE*	*p*	*B*	*SE*	*p*
Intercept	1.73**	0.10	< 0.01	−2.36	1.96	0.23	−0.14	1.37	0.92
Time (post-test)	1.86**	0.27	< 0.01	1.86**	0.27	< 0.01	−2.57	2.39	0.28
Sex (female)	–	–		0.77*	0.35	0.03	0.78*	0.35	0.03
Age	–	–		−0.01	0.02	0.58	−0.01	0.02	0.58
Years of education	–	–		0.02	0.04	0.58	0.02	0.04	0.58
Intellectual activity	–	–		0.10	0.11	0.35	0.19**	0.06	< 0.01
Physical activity	–	–		−0.16**	0.06	< 0.01	−0.05	0.04	0.24
Social activity	–	–		−0.03	0.07	0.67	−0.09*	0.04	0.02
Cumulative illness	–	–		0.24**	0.08	< 0.01	0.10*	0.04	0.02
Intellectual activity × Time	–	–		–	–		−0.19	0.16	0.24
Physical activity × Time	–	–		–	–		−0.23*	0.10	0.02
Social activity × Time	–	–		–	–		0.11	0.11	0.30
Cumulative illness × Time	–	–		–	–		0.28*	0.12	0.02
*R*^2^	0.15			0.29			0.34		

B, unstandardized regression coefficient; SE, standard error. *p < 0.05, **p < 0.01.

Notably, the outcomes of Model 1 highlighted a statistically significant correlation between the passage of time and cognitive impairment: overall, within the studied cohort, cognitive impairment worsened after 4 years (*B* = 1.86, *p* < 0.01). Meanwhile, Model 2 yielded valuable insights indicating that the initial overall severity of comorbidities and physical activity were significantly intertwined with cognitive impairment: participants with higher overall severity of comorbidities exhibited more pronounced cognitive impairment (*B* = 0.24, *p* < 0.01), whereas individuals displaying heightened levels of physical activity experienced relatively less cognitive impairment (*B* = −0.16, *p* < 0.01). The participants’ gender was found to be an additional influential predictor of cognitive impairment: women exhibited more substantial cognitive impairment (*B* = 0.77, *p* < 0.05). Lastly, the results from Model 3 indicated that individuals with elevated overall severity of comorbidities demonstrated a notably more pronounced advancement of cognitive impairment as time progressed (interaction between *Cumulative Illness* and *Time*: *B* = 0.28, *p* < 0.05). Additionally, the analysis also highlighted that individuals with higher levels of physical activity displayed a significantly attenuated progression of cognitive impairment across the four-year observation period (interaction between *Physical Activity* and *Time*: *B* = −0.23, *p* < 0.05).

In relation to specific comorbidities, Spearman’s rho correlation with cognitive impairment progression (ΔCDR-SB) was computed for each disease category. Only metabolic diseases exhibited a significant association with cognitive impairment progression, with a correlation coefficient of *rho* = 0.41 (*p* < 0.05).

## 4 Discussion

Our research supports the association between being affected by somatic conditions in old age and faster progression of impairment of cognitive functions. This association pertained to cumulative comorbidity (the overall score on the CIRS scale). When distinct categories on the scale were analysed separately, only the metabolic conditions were significantly associated with poorer outcomes with regards to cognitive functions.

The most represented conditions in the “metabolic” category in the current study were type II diabetes and dyslipidemias (hypercholesterolemia): the first was present in 24 participants and the second in 16 participants. The presence of diabetes together with amnestic mild cognitive impairment has been found to be associated with a significant increase in conversion to dementia, especially the Alzheimer’s type (Li et al., 2011, 2012). Other, later studies indicate that even just increased fasting glucose (above 100 mg/dL in individuals with MCI) is a risk factor for conversion to dementia, especially of the Alzheimer’s type, independently of whether or not amnestic-type impairments are the dominant ones (Morris et al., 2014). Recent metaanalyses support the existence of a strong relationship between having diabetes and faster progression of cognitive impairment in individuals who already show some impairment (i.e., who are diagnosed with MCI) (Cooper et al., 2015). This is in line with our results. The proposed mechanisms behind this include not only purely vascular processes but also the influence of diabetes on other neurobiological processes (Mushtaq et al., 2014; Baumgart et al., 2015). It must be noted that insulin plays a role in many important relevant processes, not least in the growth of nerve cells, the functioning of glial cells, energetic homeostasis of the brain, oxidative stress, and inflammation processes in the central nervous system (Blázquez et al., 2014; Prasad et al., 2014). The role of insulin in the maintenance of the blood–brain barrier might also be key: according to some theories, impairment of the blood–brain barrier is associated with the triggering of the so-called amyloid cascade through the initiation of inflammatory processes in the brain (Jóźwik et al., 2012; Chen et al., 2018).

The role of lipid metabolism disorders, especially those pertaining to cholesterol metabolism, in dementias have been the subject of many studies (McGuinness et al., 2009; Mushtaq et al., 2014; Baumgart et al., 2015; Pąchalska et al., 2015). Results regarding the role of cholesterol (and, more broadly, lipid metabolism) are often contradictory (McGuinness et al., 2009). It is generally accepted that there is a link between higher cholesterol levels in middle age and the risk of developing dementia at a later age (Solomon et al., 2009). On the other hand, there is much variability in the results of studies on older individuals (Gorelick et al., 2011). The current study analysed both diabetes and hypercholesterolemia as one category (“metabolic”) and thus it is impossible to say which of these factors played a bigger role in the progression of cognitive impairment.

It is somewhat surprising that no relationship was observed for hypertension, given that its role in the progression of cognitive impairment seems well documented (Reijmer et al., 2012; Baumgart et al., 2015). Results suggest that every reduction of either systolic or diastolic pressure by 10 mm hg is associated with a marked decrease in the risk of onset of dementia (Ravaglia et al., 2006). Moreover, treatment with diuretic agents has also been found to be associated with a decrease in this risk (Yasar et al., 2013). Nevertheless, in our sample, hypertension did not differentiate groups. One explanation for this may be found in newer studies which suggest that while hypertension observed around the age of 50 can be considered a risk factor for dementia, moderately increased blood pressure in older individuals is a protective factor (Duron and Hanon, 2008; Corrada et al., 2017). According to some reports, blood pressure starts to gradually decrease several years before the onset of dementia (Kahonen-Vare et al., 2004). Moreover, hypertension was a very frequent diagnosis in the studied population and including it as a dichotomized variable in our statistical model could have influenced the result. However, it should also be noted that there are reports that indicate that the influence of increased blood pressure is most pronounced when the cognitive impairment affects areas other than memory (Hansson et al., 2006).

Another variable which also differentiated individuals in terms of the severity of the progression of cognitive impairment was physical activity. Numerous studies have previously indicated that it is a factor beneficial for the prognosis of cognitive impairment (Baumgart et al., 2015; Bidzan et al., 2016; Miyawaki et al., 2017; Lipnicki et al., 2019).

On the other hand, many randomized intervention studies have failed to support the relationship between increased physical activity and slower progression of cognitive impairment (Sink et al., 2015; Andrieu et al., 2017). Research by Sabia et al. (2017) suggests that physical activity itself is not actually a protective factor, but rather decreased physical activity is an effect of the ongoing neurodegenerative process. This could be indicated by a relationship between decreased physical activity and faster progression of dementia processes in the period directly preceding the clinical manifestation of dementia, while levels of activity in earlier periods are not related to risk levels (Sabia et al., 2017). Physical activity should be considered in the context of potential risk factors for not only dementia, but also a number of other conditions, including cardio-vascular ones (which were the subject of the study). It is to be expected that comorbidities limit one’s levels of physical activity. Research by Daimiel et al. (2020) found that physical fitness, rather than physical activity itself, was associated with levels of cognitive functioning. Physical fitness was not the subject of analysis in the current study; however, it would be reasonable to assume that the presence of comorbidities could have also decreased participants’ physical activity.

### 4.1 Limitations

The lack of detailed analysis of the comorbidities, especially in terms of when they occurred, could be considered a limitation of this study. Previous literature discussing the relationship between dementia and, in particular, cardiovascular conditions has stressed their differential importance depending on the age at which they occur (Duron and Hanon, 2008). It can be expected that conditions which appear in old age have a different impact on the progression of cognitive impairment than those which are present already in middle age.

Another important limitation is the lack of detailed analysis of the treatments received by participants, both during and before observation – only the treatment being received at the time of enrollment in the study was recorded.

### 4.2 Strength of this study

The strength of this research is that we followed up for 4 years and assessed the relationship with and conversion to dementia of MCI. As previous studies have shown, metabolic diseases (DM and dyslipidemia) and physical activities are associated with progression of cognitive impairment. This investigation confirmed those findings using MCI participants living in Poland, and it may be useful for future study in this field.

## 5 Conclusion

The study was based on a four-year-long observation of individuals diagnosed with mild cognitive impairment. The results suggest poorer prognosis in terms of the progression of cognitive impairment in individuals with more comorbidities, especially metabolic conditions. This supports the particular importance of the prevention and treatment of somatic illness for decreasing the prevalence of dementia. Further studies should analyse the types of treatments being undertaken for such comorbidities and their effectiveness in individuals with mild cognitive impairment.

## Data availability statement

The raw data supporting the conclusions of this article will be made available by the authors, without undue reservation.

## Ethics statement

The studies involving humans were approved by Bioethics Committee at the Medical University of Gdansk, Poland. The studies were conducted in accordance with the local legislation and institutional requirements. The participants provided their written informed consent to participate in this study. Written informed consent was obtained from the individual(s) for the publication of any potentially identifiable images or data included in this article.

## Author contributions

LB, IB-B, MB-W, MB, MO, and PJ: conceptualization, formal analysis, and writing—original draft. LB, IB-B, MB-W, and MB: data curation and investigation. LB: funding acquisition and supervision. LB, PJ, and MO: methodology. LB and MB: project administration. LB, MB, and PJ: writing—review & editing. All authors contributed to the article and approved the submitted version.
